# Autochthonous Infections with Hepatitis E Virus Genotype 4, France

**DOI:** 10.3201/eid1808.111827

**Published:** 2012-08

**Authors:** Philippe Colson, Pauline Romanet, Valérie Moal, Patrick Borentain, Raj Purgus, Alban Benezech, Anne Motte, René Gérolami

**Affiliations:** Institut Hospitalo-Universitaire Méditerranée Infection, Marseille, France (P. Colson, P. Romanet, A. Motte);; Aix-Marseille Université, Marseille (P. Colson);; Hôpital Conception, Marseille (V. Moal, R. Purgus);; and Centre Hospitalo-Universitaire Conception, Marseille (P. Borentain, A. Benezech, R. Gérolami)

**Keywords:** hepatitis E virus, hepatitis E, genotype 4, France, viruses, foodborne infection, zoonosis, pig, biosurveillance

## Abstract

During January–March 2011, diagnoses of hepatitis E virus (HEV) infection increased in Marseille University hospitals in southeastern France. HEV genotype 4, which is described almost exclusively in Asia, was recovered from 2 persons who ate uncooked pork liver sausage. Genetic sequences were 96.7% identical to those recently described in swine in Europe.

In industrialized countries, most cases of hepatitis E virus (HEV) infection in humans are autochthonous (*1*). Pigs are a major reservoir of HEV, and transmission of virus to humans who ate raw or undercooked pork has been reported (*1**–**3*). In France, >300 cases of HEV infection are reported annually (*3*); most infections are autochthonous and occur in southern France, where the prevalence of anti-HEV IgG is higher than in northern France (*3**–**5*). Almost all cases of autochthonous HEV infection reported in Europe have involved genotype 3 strains (*1**–**6*).

Beginning February 21, 2011, a biosurveillance program (EPIMIC) (*7*) detected an increase in the number of HEV infections diagnosed at Marseille University hospitals in southeastern France. During February 21–March 28, the weekly number of serum samples that were tested and found positive for HEV was above the elected critical threshold (Technical Appendix Figure 1).

## The Cases

In Marseille during January–March 2011, a total of 11 cases of HEV infection were confirmed by anti-HEV IgM testing and detection of HEV RNA in serum samples. EIAgen assays (Adaltis Italia, Casalecchio di Reno, Italy) were used to detect anti-HEV IgM and IgG. Additional anti-HEV IgM testing was performed by using the Assure HEV IgM Rapid Test (MP Biomedicals, Illkirch, France) and the *recom*Line HEV IgG/IgM test (Mikrogen Diagnostik, Neuried, Germany). HEV RNA was detected by using a real-time reverse transcription PCR targeting open reading frame (ORF) 2 (*2*).

The mean age of the case-patients was 57 years (±11 years). Of the 11 case-patients, 10 were male and 3 were kidney transplant recipients (Table 1, Table 2). HEV infection was clinically asymptomatic in all transplant recipients; the infection was diagnosed after routine posttransplant laboratory tests showed elevated levels of liver enzymes. Longitudinal testing indicated chronic HEV infection in 1 case-patient (no. 5), and an 80-year-old case-patient died 9 weeks after disease onset.

**Table 1:** Epidemiologic and virological findings for case-patients and contacts with HEV infection, Marseille, France, January–March 2011*

Case-patients and contacts, by ID no.	Day obtained, serum sample (retrospectively tested sample)	Anti-HEV IgG EIA result†	Anti-HEV IgM result, by testing method			HEV RNA in serum (C_t_)	HEV genotype
			EIA†	Rapid test‡	Immunoblot		
1	Feb18	10.3	14.2	Positive	Positive	Positive (29)	4
1a§	*Mar 2*	*11.4*	*0.2*	*Negative*	*Not tested*	*Positive (38)*	NA
1a§	*Mar 16*	*>10.0*	*2.0*	*Not tested*	*Borderline*	*Negative*	NA
1b¶	*Mar 2*	*Negative*	*Negative*	*Negative*	*Negative*	*Negative*	NA
2	Mar 21 (Feb 7)	11.2	15.6	Positive	Positive	Positive (28)	4
2a#	*Mar 21*	*6.3*	*15.6*	*Positive*	*Positive*	*Negative*	NA
2b**	*Mar 21*	*0.1*	*Negative*	*Negative*	*Negative*	*Negative*	NA
3	Jan 4	10.7	16.6	Positive	Not tested	Positive (27)	3f
4	Feb 3 (Jan 6)	5.2	6.3	Weakly reactive††	Positive	Positive (26)	3c
5‡‡	Feb 3	0.1	1.6	Weakly reactive	Negative	Positive (31)	3f
6	Mar 1	11	14	Positive	Positive	Positive (38)	NA
7	Mar 2	10.6	15.1	Positive	Negative	Positive (27)	3c
8‡‡	Mar 9	11.1	14.9	Positive	Not tested	Positive (28)	3c
9	Mar 18	10.5	16.4	Positive	Not tested	Positive (28)	3f
10‡‡	Mar 21	0.3	15.6	Positive	Not tested	Positive (24)	3c
11	Mar 23	1.2	13.8	Positive	Positive	Positive (27)	3f

*Italics indicate results for contacts of case-patients. HEV, hepatitis E virus; ID, identification; EIA, enzyme immunoassay; C_t_, cycle threshold; NA, not applicable. †EIA score represents test/cutoff optical density ratios. ‡An immunochromatographic rapid test. §Contact (wife) of case-patient no. 1. ¶Contact (friend) of case-patient no. 1. #Contact (friend) of case-patient no. 2. **Contact (sister) of case-patient no. 2. ††Tested on a serum sample collected on March 3, 2011. ‡‡Kidney transplant recipient.

**Table 2:** Epidemiologic and clinical findings for case-patients and contacts with HEV infection, Marseille, France, January–March 2011*

Case-patients and contacts, by ID no.	Age, y/sex	Clinical signs	ALT, IU/L†	Consumed uncooked pig liver sausage
1	62/M	Asthenia, jaundice, nausea	2,124	Yes
1a‡	*57/F*	*None*	*165*	*Yes*
1b§	*60/M*	*None*	NA	*Yes*
2	49/M	Asthenia, jaundice	138	Yes
2a¶	*46/F*	*None*	*40*	*Yes*
2b#	*51/F*	*None*	*40*	*Yes*
3	45*/F*	Jaundice	1,696	Yes
4	80/M	Asthenia, jaundice	1,885	No
5**	63/M	None	340	No
6	68/M	Asthenia, fever, jaundice	1,135	Yes
7	54/M	Asthenia, jaundice	88	No
8**	58/M	None	21	No
9	38/M	Asthenia, fever, dark urine	1,554	Yes
10**	52/M	None	71	Yes
11	58/M	Asthenia, jaundice	2,500	No

*Italics indicate features for contacts of case-patients. HEV, hepatitis E virus; ID, identification; ALT, alanine aminotransferase; NA, not available. †Upper limit of normal, 40 IU/L. ‡Contact (wife) of case-patient no.1. §Contact (friend) of case-patient no.1. ¶Contact (friend) of case-patient no.2. #Contact (sister) of case-patient no.2. **Kidney transplant recipient.

HEV 5′-ORF2 RNA was recovered from the serum of 8 case-patients. Phylogenetic analysis (*2*) showed that 4 patients each had HEV genotype 3c or 3f (Technical Appendix Figure 2). Sequences from 2 unrelated case-patients (nos. 7, 8) showed 99.8% identity; for other pairwise comparisons, maximal identity was 93.4% (mean 83.6%).

We could not recover HEV 5′-ORF2 RNA from the serum of 3 case-patients (nos. 1, 2, 6). For case-patient 6, this could be explained by a low viral load. When targeting ORF1 and 3′-ORF2, we recovered HEV genotype 4 (HEV-4) from serum samples from case-patients 1 and 2 (Figure; Technical Appendix Figure 3) (*5**,**10*). Molecular testing results for these samples were checked in duplicate and by testing different serum samples from the same case-patients. HEV-4 RNA from the 2 case-patients showed 100% identity for ORF1 and 99.8% identity for ORF2. The next closest match (96.7% identity) was the first HEV-4 RNA in swine reported in Europe (GenBank accession no. HQ857384) (Figure) (*8*). The next 2 closest matches (91.0%–91.3% identity) were recovered from swine in China (GenBank accession nos. EU676172 and DQ279091) (Figure). However, the sequences showed only 86.0% identity with a genotype 4f HEV RNA recovered from a patient in Germany (*9*).

**Figure:**
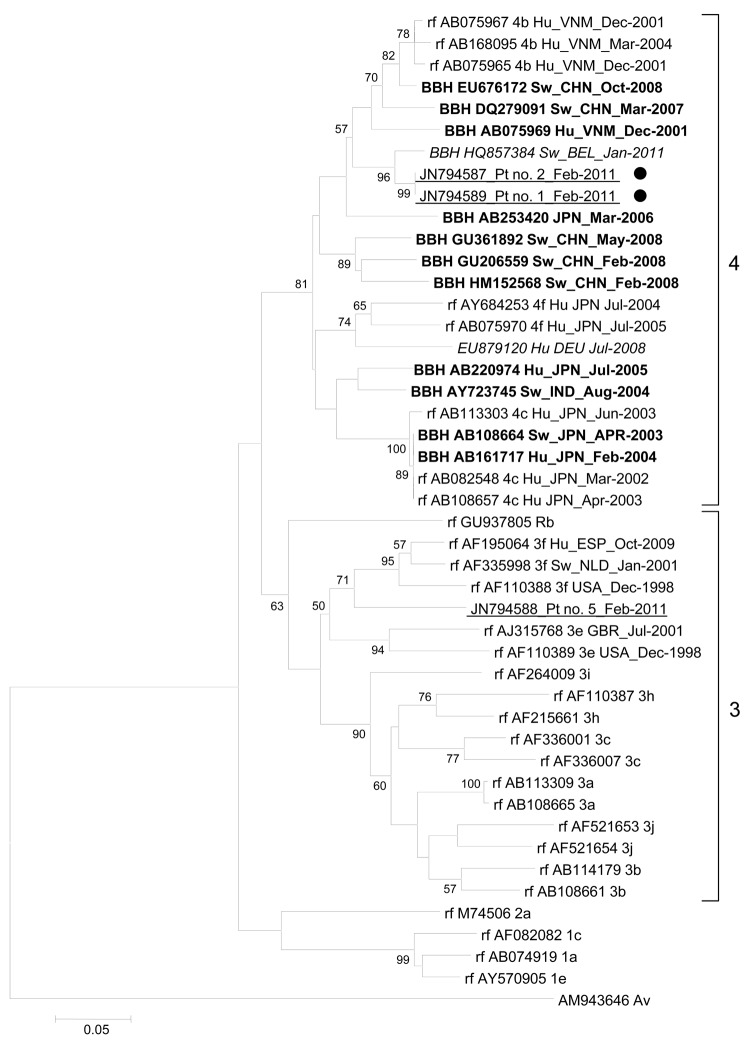
Phylogenetic tree based on partial (186 nt) sequence of the 5′ end of open reading frame 1 of the hepatitis E virus (HEV) genome (nt 133–318; GenBank accession no. AB291961). **Boldface** indicates sequence with the highest BLAST scores (http://blast.ncbi.nlm.nih.gov/Blast.cgi); red (*italics*) indicates sequences obtained from a swine in Belgium with HEV genotype 4 (*8*) and a human in Germany with autochthonous HEV infection (*9*); blue (underlining) indicates sequences obtained from humans in Marseille, France, during January–March 2011; black dots indicate patients who ate uncooked pig liver sausage. Reference sequences (rf) with known genotypes and subtypes are indicated (*6*). Numbers on right indicate genotype. For sequences from this study, nucleotide alignments were performed by using ClustalX version 2.0 (www.clustal.org/download/current). The tree was constructed by using MEGA4 (www.megasoftware.net) and the neighbor-joining method as described (*2*). Branches were obtained from 1,000 resamplings of the data; those with bootstrap values >50% are labeled on the tree. The avian HEV sequence AM943646 was used as an outgroup. Scale bar indicates nucleotide substitutions per site. HEV sequences are labeled with GenBank accession number, host, country where isolated, and collection or submission date. Hu, human; VNM, Vietnam; BBH, best BLAST hit; Sw, swine; CHN, China; BEL, Belgium; Pt, patient; JPN, Japan; DEU, Germany; IND, India; Rb, rabbit; ESP, Spain; NLD, the Netherlands; USA, United States; GBR, United Kingdom; Av, avian.

None of the 11 case-patients had traveled abroad during the 2–9 weeks before hepatitis onset. Of the 11 case-patients, 6 (55%), including the 2 with HEV-4, ate uncooked pig liver sausage (*figatelli* [*2*]) (Table 2). The 2 patients with HEV-4 were not related. Because 4 contacts of the 2 HEV-4–infected case-patients also ate some of the figatelli eaten by the case-patients (Table 1, Table 2), we investigated whether these contacts had markers for HEV infection. Test results for anti-HEV IgM were positive for the wife of case-patient 1 and a friend of case-patient 2; the HEV PCR result was barely positive for contact 1a, who had an elevated level of alanine aminotransferase (165 IU/L; reference <45 IU/L). These 2 persons were clinically asymptomatic.

## Conclusions

We report a concurrent rise in testing for and diagnoses of HEV infections in Marseille during January–March 2011. This rise may reflect increased clinician awareness of HEV or an increased incidence of HEV infection that may have resulted from the use of improved tools for HEV diagnosis.

In industrialized countries, HEV has increasingly been recognized as a possible cause of hepatitis in diverse clinical settings, including in solid-organ transplant recipients. In our study, 3 (27%) of the case-patients were solid-organ transplant recipients. This number suggests a high incidence of HEV infection among transplant recipients in the geographic area we studied, and it is consistent with the incidence (≈3.2/100 person-years) of HEV infection among organ transplant recipients in Toulouse in southwestern France (*4*). Liver biochemical testing is routinely performed after organ transplantation; such testing enabled the detection of subclinical HEV infection in the 3 transplant recipients reported here. Subclinical HEV infection can also occur in immunocompetent persons (*1**,**11**,**12*); thus, we question whether the incidence of HEV infection among transplant recipients in southern France might reflect that in the general population. Moreover, 52% of adult blood donors sampled in southwestern France in 2003–2004 were retrospectively shown to be HEV seropositive by using a newly available assay (*4*), and HEV was retrospectively shown to be an underdiagnosed cause of liver injury in the United States and United Kingdom (*13*).

A considerable proportion of HEV infections in southern France may be linked to the consumption of uncooked figatelli, which has been identified as a source of zoonotic foodborne HEV transmission (*2**,**4*). In our study, 55% of the HEV-positive patients reported eating uncooked figatelli, and in another study in France, 2 HEV-positive patients reported eating uncooked figatelli (*11*). These cases indicate insufficient prevention of HEV transmission to humans through uncooked figatelli consumption (*2*).

The major finding in our study was the recovery of HEV-4 sequences from 2 of 6 case-patients who ate uncooked figatelli purchased in southern France. These sequences were closely related to the sequence for the first-reported HEV-4 RNA in swine in Europe (*8*), which was recovered in Belgium and classified as genotype 4b. HEV-4 is indigenous to Asia, where it has been recovered from pigs and humans (*1**,**6**,**14*). For the 2 HEV-4 RNA sequences obtained in our study, the best matches among sequences from Asia were from HEV strains from swine in China. Since its first report in China in 1999, HEV-4 has been increasingly described as endemic in pigs and involved in most sporadic cases of hepatitis E (*1**,**14*). Several HEV subtypes have been described in China, including subtype 4b (*1**,**6**,**14*). In Europe, autochthonous infection with a genotype 4f HEV in a person in Germany was reported in 2008 (*9*), and a case of HEV-4 infection acquired in India was reported for a person in England in 2010 (*15*). Studies in China have shown relatedness between HEV-4 sequences recovered from humans and swine in the same geographic area, which suggests zoonotic transmission (*1*). Moreover, sequences for HEV-4 RNA recovered from a father and son in Japan who ate barbecued pig meat were almost identical, and sequences were identical for HEV-4 RNA from packaged pig liver sold in a grocery store and from a patient in whom HEV infection developed after the person ate grilled pig liver (*1**,**2*).

Together with previous findings, our results bring up the question as to whether HEV-4 was introduced into domestic pigs or whether pig meat of Asian origin was introduced into France. Moreover, it prompts a study of whether HEV-4 strains circulate and spread in Europe as an effect of globalization of HEV zoonosis.

Technical Appendix Monitoring of serum samples tested and positive anti–hepatitis E virus (HEV) IgM and HEV RNA test results and phylogenetic trees based on a partial sequence of open reading frame 2 and a partial nucleotide sequence of the 3′ end of open reading frame 2 of the HEV genome.
