# Supplementary material for: Autochthonous Infections with Hepatitis E Virus Genotype 4, France
Source: Emerg Infect Dis. 2012 Aug;18(8):1361–4. doi: 10.3201/eid1808.111827 (PMC3414032; doi:10.3201/eid1808.111827)
Supplement: Technical Appendix — Monitoring of serum samples tested and positive anti–hepatitis E virus (HEV) IgM and HEV RNA test results and phylogenetic trees based on a partial sequence of open reading frame 2 and a partial nucleotide sequence of the 3′ end of open reading frame 2 of the HEV genome. [file 11-1827_Techapp-s1.pdf]

Article DOI: http://dx.doi.org/10.3201/eid1808.111827

Autochthonous Infections with Hepatitis E Virus Genotype 4, France

Technical Appendix

Viral hepatitis		Anti-HEV IgM+ Nb of samples	Anti-HEV IgM+ Diagnoses (nb	Anti-HEV IgM+ Diagnoses (%)	PCR-documented autochthonous hepatitis E Diagnoses (nb
Nb weeks		298	296	296	262
Total		6 646	355	5,3	107
Estimated nb/y		1 160	62	5,4	21
Per week:					
Median		22	1,0	4,5	0,0
Mean		22	1,2	5,4	0,4
2 SD		30	3,0	9,9	1,2
Elected high critical threshold		52	4,2	8,0	1,6
13/12/2010	424	42	2	5	0
20/12/2010	425	37	2	5	0
27/12/2010	426	23	2	9	0
03/01/2011	427	42	3	7	1
10/01/2011	428	33	2	6	0
17/01/2011	429	41	1	2	0
24/01/2011	430	42	2	5	1
31/01/2011	431	53 ▲	2	4	1
07/02/2011	432	51	2	4	0
14/02/2011	433	50	4	8	1
21/02/2011	434	54 ▲	5 ▲	9	0
28/02/2011	435	62 ▲	10 ▲	16	2 ▲
07/03/2011	436	62 ▲	5 ▲	8	1
14/03/2011	437	48	5 ▲	10	1
21/03/2011	438	46	7 ▲	15	3 ▲
28/03/2011	439	47	5 ▲	11	0
04/04/2011	440				

Technical Appendix Figure 1. Weekly monitoring of the numbers of serum samples tested for hepatitis E virus (HEV) and the number with positive anti-HEV IgM and HEV RNA test results during December 13, 2010–April 4, 2011. Data are entered into a Microsoft Excel file each week (*1*). EPIMIC, a biosurveillance program (*1*), automatically and instantly calculates the mean value and the standard deviation (SD) for all entered datasets. Any significant increase beyond the critical threshold, defined by the mean value +2 SDs, generates a red signal. Values that exceed the critical threshold are indicated in the figure by a black triangle and red font.

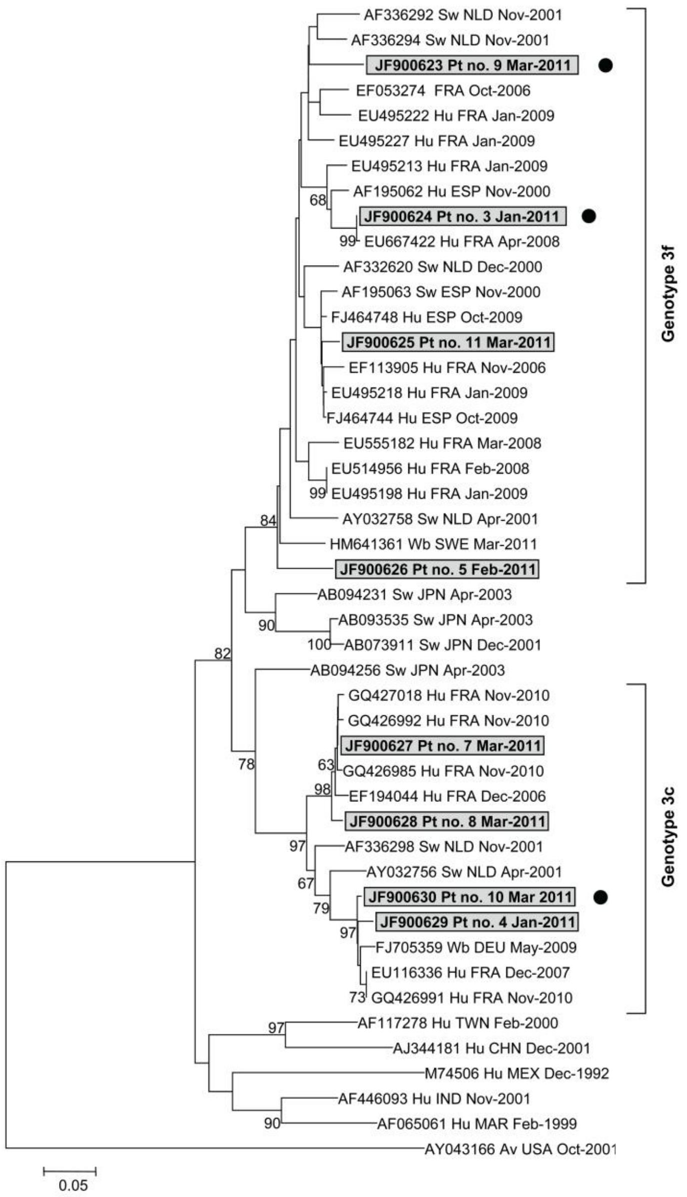

Technical Appendix Figure 2. Phylogenetic tree based on partial (204-nt) sequence of open reading frame 2 of the hepatitis E virus (HEV) genome (nt 6066–6269; GenBank accession no. AB291961). The phylogenetic analysis is as described in the Figure, except that avian (Av) HEV sequence AY043166 was used as an outgroup. Sequences obtained from humans in Marseille, France, during January–March 2011, are shown in boxes with gray background; black circles indicate patients who ate uncooked pig liver sausage. Genotype is indicated on the right. The scale bar indicates the number of nucleotide substitutions per site. HEV sequences are labeled with GenBank accession number, host, country where isolated, and collection or submission date. CHN, China; DEU, Germany; ESP, Spain; FRA, France; Hu, human; IND, India; JPN, Japan; MAR, Marseille; MEX, Mexico; NLD, the Netherlands; Sw, swine; SWE, Sweden; TWN, Taiwan; USA, United States; Wb, wild boar.

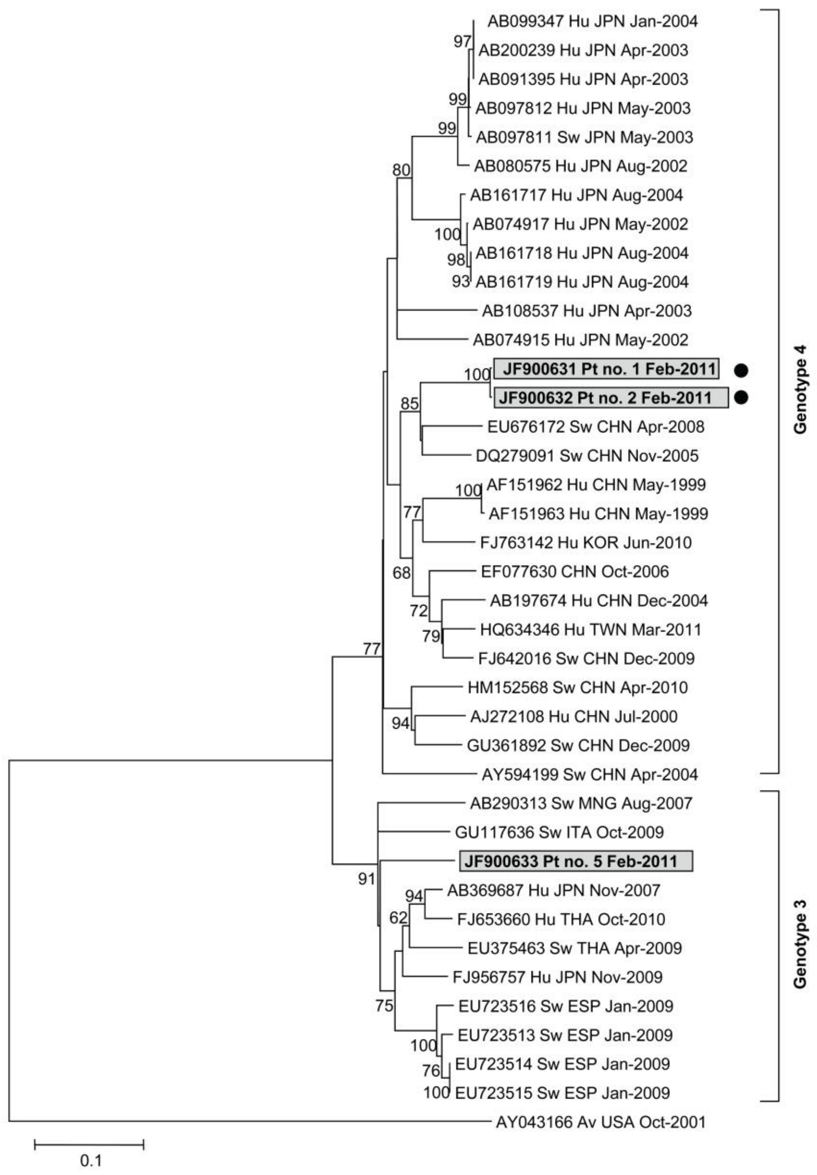

Technical Appendix Figure 3. Phylogenetic tree based on partial (537 nt) nucleotide sequence of the 3′-end of open reading frame 2 of the hepatitis E virus (HEV) genome (nt 6439–6975; GenBank accession no. AB291961). The phylogenetic analysis is as described in the Figure, except that avian (Av) HEV sequence AY043166 was used as an outgroup. Sequences obtained from humans in Marseille, France, during January–March 2011, are shown in boxes with gray background; black circles indicate patients who ate uncooked pig liver sausage. Genotype is indicated on the right. The scale bar indicates the number of nucleotide substitutions per site. HEV sequences are labeled with GenBank accession number, host, country where isolated, and collection or submission date. CHN, China; ESP, Spain; Hu, human; ITA, Italy; JPN, Japan; KOR, South Korea; MNG, Mongolia; Sw, swine; THA, Thailand; TWN, Taiwan; USA, United States.

Reference

1. Parola P, Colson P, Dubourg G, Million M, Charrel R, Minodier P, et al. Letter to the Editor. Group A streptococcal infections during the seasonal influenza outbreak 2010/11 in South East England. Euro Surveill. 2011;16:pii=19816. PubMed
